# The Roles of Trust and Its Antecedent Variables in Healthcare Consumers’ Acceptance of Online Medical Consultation during the COVID-19 Pandemic in China

**DOI:** 10.3390/healthcare11091232

**Published:** 2023-04-26

**Authors:** Mian Yan, Meijuan Zhang, Alex Pak Ki Kwok, Haoyan Zeng, Yanfeng Li

**Affiliations:** 1 School of Intelligent Systems Science and Engineering, Jinan University, Zhuhai 519070, China; 2 GBA and B&R International Joint Research Center for Smart Logistics, Jinan University, Zhuhai 519070, China; 3 School of Management, Jinan University, Guangzhou 510000, China; 4 Data Science and Policy Studies Programme, Faculty of Social Science, The Chinese University of Hong Kong, Hong Kong 999077, China

**Keywords:** online medical consultation, public health, technology acceptance, trust

## Abstract

Online medical consultation (OMC) is generating considerable interest among researchers and practitioners due to the mandatory quarantine measures implemented during the COVID-19 pandemic in China. However, the acceptance rate of OMC has declined over time. This paper aims to empirically investigate OMC acceptance using a proposed research model by integrating the technology acceptance model (TAM) with trust and its antecedent variables. A quantitative self-administered cross-sectional survey was conducted to collect data from 260 healthcare consumers. A partial least squares structural equation modeling method was used to examine the data. Results revealed that healthcare consumers’ behavioral intention was influenced by attitudes, while perceived usefulness and trust significantly influenced behavioral intention through attitude as a mediator. In addition, perceived risk, perceived privacy protection, network externalities, cognitive reputation, and interactivity directly influenced trust. Overall, the research model explained 50% of the variance in attitude and 71% of the variance in behavioral intention. The study’s findings should provide useful insights into making effective design, development, and implementation decisions for OMC services.

## 1. Introduction

The increasing demand for medical services, particularly among aging populations, has created a global supply-demand imbalance in healthcare systems, leading to challenges such as unaffordability, inequality, and inefficiency [1]. Health information technologies (HITs) have emerged as potential solutions to these challenges, as they can enhance the quality, efficiency, and fairness of healthcare services. HIT encompasses a wide range of products, goods, and services, including medical equipment, electronic health records (EHRs), assistive technology and sensors, mobile health technologies, and telehealth [2]. One of the latest innovations to address the growing medical needs is online medical consultation (OMC) [3,4]. OMC is gaining popularity as a convenient and flexible alternative to traditional face-to-face consultations, overcoming time and space constraints. It has become a global trend, accounting for a significant portion of the telemedicine market [5]. This sector focuses exclusively on remote consultations between patients and physicians via websites or mobile applications. Both public and private medical institutes can organize these consultations. OMC is anticipated to generate US$25.31 billion in revenue worldwide by 2023, with an annual growth rate of 8.76% [6].

The COVID-19 pandemic has increased the number of people trying OMC in China due to mandatory quarantine measures and government policies [7]. Nevertheless, the public’s adoption of OMC is still relatively low in China compared to more developed countries [8]. It is unclear whether the adoption rate of OMC can continue to increase after the pandemic [9]. Therefore, further research is required to determine what factors contribute to OMC’s acceptance and usage intention among healthcare consumers (i.e., patients or people with healthcare concerns) in order to improve public health outcomes. This issue relates to the adoption, use, and acceptance of new technology, which can be explained using models and theories such as the Technology Acceptance Model (TAM) [10,11,12], the Diffusion of Innovation (DOI) [13], and the Unified Theory of Acceptance and Use of Technology (UTAUT) [14,15]. Among them, TAM is one of the most widely researched models in information science [16], proposing a causal link between belief, attitude, intention, and behavior to understand the adoption of technology. It includes two key factors, perceived ease of use and perceived usefulness, to explain user acceptance of new technologies.

Numerous studies have examined the influence of information technology on healthcare quality, efficiency, and cost [17,18]. However, these studies mainly focused on service provider-centric design and execution [16]. Existing research on healthcare consumers’ perceptions of technology and its relationship to behavior is limited. Compared to other revolutionary technology applications, such as virtual reality, the OMC is a health and life-related application requiring more prudence with its service [19]. Users may have concerns regarding diagnostic accuracy and may be wary about disclosing sensitive personal data online due to privacy concerns [20]. Furthermore, OMC may hinder people from directly interacting with physicians, inhibiting healthy doctor-patient relationships [21]. Notably, these concerns arise from the lack of trust in various aspects of OMC. Humans need trust, especially when making big, risky decisions [22]. Given the potential risks (e.g., privacy-, security-, and information quality-related risks) associated with the usage of OMC platforms, it is necessary to understand the influence of trust in the platforms’ usage intention.

According to a recent review article [17], little research has extended the original TAM model to incorporate trust and its antecedent variables to better understand why healthcare consumers use OMC services. In this context, service providers and users must understand the impact of trust and its antecedent factors on OMC acceptability and usage. Thus, this study contributes to a rigorous empirical analysis of OMC adoption from a healthcare consumer-centric (vs. provider-centric) and trust viewpoint. It aims at answering the following research questions:

RQ1. How does trust affect healthcare consumers’ adoption of OMC?

RQ2. What are the influences of antecedent variables on healthcare consumers’ trust formation in the acceptance of OMC?

This study aims to provide evidence and discussions on the operation and development of OMC from a healthcare consumer-centric and trust viewpoint. As trust is an important factor in technology acceptance [23], the study aims to explore the factors influencing trust formation among healthcare consumers when using OMC. By doing so, the study seeks to provide evidence and insights into how OMC can be developed and operated in a way centered on the needs and perspectives of healthcare consumers, with a particular emphasis on building trust. Ultimately, the study aims to contribute to the ongoing development of OMC as a valuable and trusted tool for delivering healthcare services. The following sections comprise the remainder of this article. Section 2 discusses the theoretical foundations and model assumptions. Section 3 introduces the research methodology. Section 4 illustrates an empirical test. Section 5 presents a discussion of the study’s results and implications. Finally, Section 6 is the study’s conclusions.

## 2. Theoretical Development and Research Model

This section aims to conduct a literature review on OMC adoption and propose a TAM-based research model to examine the predictive relationship between trust and its antecedent variables on healthcare consumers’ willingness to use OMC.

### 2.1. Online Medical Consultation and Its Adoption

Information technology has changed how health information is gathered and used. OMC is a kind of online healthcare technology offered by qualified healthcare experts via third-party digital platforms [9]. Prior research on OMC has primarily focused on the effects of service and physician characteristics on patients’ physician selection processes. For example, Littlejohns et al. [24] examined the factors that patients consider when selecting a physician for consultation via an OMC platform and the effect of these factors on physicians’ consultation volumes. Chiu et al. [25] explored the determinants of consulting prices using OMC. However, according to our systematic literature review, few studies have examined healthcare consumers’ trust and its antecedent variables in-depth to understand why they would use OMC (see Table 1). To this end, this study responded to the need to understand how healthcare consumers’ trust and its antecedent variables can influence the known determinants of IT adoption and use, e.g., attitude and behavioral intention. It proposed a research model based on TAM to examine the predictive relationships between trust and its antecedent variables on healthcare consumers’ intention to seek medical advice online. Based on a thorough literature search, our research model examines the effects of perceived usefulness, perceived ease of use, attitude, trust, interactivity, perceived risk, perceived privacy protection, cognitive reputation, and network externalities on behavioral intention. Each variable is defined below, along with 18 hypotheses.

### 2.2. The Base Model

OMC is a relatively new Internet-based medical information system. As a result, the technology acceptance model (TAM) [40,41] was employed as the base model to account for users’ behavioral intention to use this newly produced technology. TAM is a frequently used theoretical model for assessing technology adoption in information systems [16] and diverse types of e-Health applications [42].

#### 2.2.1. Attitude (ATT) and Behavioral Intention (BI)

TAM has two major endogenous variables: attitude and behavioral intention. In this study’s context, ATT refers to the subjective pleasant or bad sentiments users experience when using OMC [40]. Whereas BI refers to the degree to which users intend to use OMC [40]. Razmak and Bélanger [42] noted that individuals’ attitudes about health information technology significantly impact their adoption. Therefore, it is believed that if healthcare consumers had a positive attitude towards OMC, they would be more inclined to use it for healthcare purposes. Consequently, the following hypothesis was developed:

**H1.**  *Attitude will have a significant positive effect on behavioral intention*.

#### 2.2.2. Perceived Usefulness (PU) and Perceived Ease of Use (PEOU)

TAM suggested that an individual’s attitude toward using a new system is determined by their salient beliefs about the system, specifically, perceived usefulness and perceived ease of use [40]. In this study, PU refers to how people feel OMC could enhance their health conditions [40]. PEOU refers to the extent healthcare consumers think utilizing OMC would be free of effort, positively influencing perceived usefulness and attitude [40]. Jin et al. [43] found that perceived usefulness and perceived ease of use have a significant positive effect on mHealth app use. Wang et al. [44] revealed that PEOU has a significant effect on PU, and PU significantly affects the patients’ attitudes and behavioral intentions toward mobile medical platforms. AlBar and Hoque [45] found that PEOU and PU significantly influenced attitudes towards e-health services, and PEOU positively impacts PU. Consequently, the following hypothesis was developed:

**H2.**  *Perceived usefulness will have a significant positive effect on attitude*.

**H3.**  *Perceived usefulness will have a significant positive effect on behavioral intention*.

**H4.**  *Perceived ease of use will have a significant positive effect on perceived usefulness*.

**H5.**  *Perceived ease of use will have a significant positive effect on attitude*.

### 2.3. Extending TAM with the Inclusion of Trust and Its Antecedent Variables

#### 2.3.1. Trust (TRU)

While using OMC services, healthcare consumers must grant service providers access to their personal information and historical health data. They receive treatment or medical advice based on healthcare professionals’ online diagnoses. In this process, trust is of great importance, especially for consumers to disclose personal information to the OMC platforms and service providers. In other words, the extent to which a healthcare consumer trusts OMC (including the platform and healthcare professionals) determines whether they may accept and adopt OMC services. Consequently, trust in this study context is considered a core component for successful OMC implementations. The present study defines trust as a psychological state that involves the willingness to accept the vulnerability of OMC services based on positive expectations of another’s behavior or intentions [46]. Trust is especially crucial where there is uncertainty and a lack of regulation [47]. It has been recognized as the primary basis for consumers to make purchase decisions online without sufficient information [48]. Bozic [49] noted that consumers’ confidence in service providers underpins their loyalty, long-term partnerships, commitment, and product acceptance. If service providers fail to deliver trustworthiness, consumers are unlikely to complete a transaction [16]. Numerous studies have demonstrated that people’s intention to use technology is influenced by their trust in the technology platforms and service providers. For instance, Beldad and Hegner [50] showed that customers’ trust in the fitness app developer affects their perception of the app’s usefulness, ultimately leading to intent to use. Kamal et al. [51] discovered that individuals’ intention to use telemedicine services is influenced by their level of trust. Therefore, it is reasonable to anticipate that if healthcare consumers trust that the OMC service providers can satisfy their needs, they will be more likely to view the OMC services as useful and beneficial, leading to acceptance. Consequently, the following hypotheses were formed:

**H6.**  *Trust will have a significant positive effect on perceived usefulness*.

**H7.**  *Trust will have a significant positive effect on attitude*.

**H8.**  *Trust will have a significant positive effect on behavioral intention*.

#### 2.3.2. Network Externalities (NE)

NE refers to the occurrence in which healthcare consumers’ perceived value of OMC rises as the number of other users who use the service grows [52]. Network externality is particularly prevalent in the communications industry or network products, where users’ perceived value of the service or product increases as the number of users in the network grows [53]. Miao et al. [1] reported that users prefer mHealth technologies with more extensive user networks over traditional healthcare services. It is also claimed that network externalities can impact consumers’ perceived usefulness and acceptance of a service [53,54]. It is speculated that, on the one hand, healthcare consumers will be more likely to form trust in OMC platforms and service providers when there are more users in the network. On the other hand, the adoption rate of OMC among healthcare providers may grow with the increased need from consumers, which, in turn, benefits healthcare consumers by reducing OMC waiting time and providing more choices while maintaining high service quality. Therefore, the following hypotheses were made:

**H9.**  *Network externalities will have a significant positive effect on trust*.

**H10.**  *Network externalities will have a significant positive effect on perceived usefulness*.

#### 2.3.3. Interactivity (INT)

The defining characteristic of OMC is the potential for instant communication with healthcare professionals. Therefore, INT is embedded into the proposed model. INT, which denotes the exchange of communication between service providers and users, plays a crucial role in the context of information and communication technologies and online platforms [55]. Online interactivity is particularly vital when face-to-face interaction with service providers is not possible, as it helps enhance consumer engagement in online purchasing scenarios [56]. Healthcare consumers in OMC environments may experience uncertainty and concern regarding OMC service and platform quality since they cannot physically see the physicians and visit the service providers. Therefore, effective and efficient interaction (two-way communication) between healthcare consumers and service providers is crucial for inspiring consumers’ trust and confidence in OMC services. Bao et al. [57] showed that perceived interactivity improves e-commerce marketplace trust. Khare et al. [55] also noted that website interactivity is crucial for attracting and retaining clients. Pituch and Lee Pituch and Lee [58] also suggested that increased two-way communication can lead to easier usage of an information system. Consequently, the following hypothesis was developed:

**H11.**  *Interactivity will have a significant positive effect on trust*.

**H12.**  *Interactivity will have a significant positive effect on perceived ease of use*.

#### 2.3.4. Perceived Privacy Protection (PPP)

Privacy is always a concern when sharing information online, especially in healthcare services [59]. Privacy risk is the potential exposure of a user’s private information [60]. PPP relates to the perceptions of privacy risk reduction in the usage of OMC services. As OMC services demand a considerable variety of personal information, such as phone numbers, disease history, and residence addresses, privacy is another primary concern regarding OMC adoption. Esmaeilzadeh [61] showed a positive relationship between users’ trust in their capabilities and perceived transparency of health information exchange privacy policies. Similarly, Cheng and Mitomo [62] found that privacy concerns about the use of smart wearable devices had a significant impact on people’s perceived usefulness of applications. Based on these findings, the following hypotheses were developed:

**H13.**  *Perceived privacy protection will have a significant positive effect on trust*.

**H14.**  *Perceived privacy protection will have a significant positive effect on perceived usefulness*.

#### 2.3.5. Perceived Risk (PR)

PR refers to the uncertainty and adverse outcomes customers anticipate [63]. Previous research has categorized risks into six areas: performance, financial, social, psychological, safety, and opportunity/time [64]. This present study considered perceived risk as the financial, safety, and performance risks healthcare consumers may experience while using OMC services. The literature has long history showing that trust and perceived risk are closely related. For instance, Park et al. [65] found that the perceived risk associated with using mobile payment had a negative impact on consumers’ trust in such payment systems. Kwok et al. [66] discovered that perceived risk influences public compliance with COVID-19 health interventions. Additionally, PR influences customers’ decision-making [67]. It may hinder customers from trying new technologies [68]. Qi et al. [3] found that perceived risk negatively affects perceived usefulness, which significantly influences public intention to use e-consultation. As OMC is a healthcare technology directly tied to healthcare consumers’ health, if healthcare consumers perceive risks associated with OMC, they would question if OMC can help them solve problems and provide them with benefits. Therefore, this study hypothesized that:

**H15.**  *Perceived risk will have a significant negative effect on trust*.

**H16.**  *Perceived risk will have a significant negative effect on perceived usefulness*.

#### 2.3.6. Cognitive Reputation (CR)

CR quantifies the trustor’s (i.e., healthcare consumer’s) level of cognitive acquaintance with the trustee (i.e., OMC service providers) [69]. When healthcare consumers lack direct knowledge or actual connection with an information technology service, they will attempt to create cognitive familiarity based on relevant secondary information, i.e., the goodwill of the platform [69]. Literature indicates that reputation directly affects trust [70]. Costantino et al. [71] also demonstrated that patient confidence in gastroenterology tele-visits is determined by the service provider’s reputation during COVID-19. Additionally, Wu and Chen [72] also demonstrated that reputation favors the perceived usefulness of online platforms. In the context of OMC, it is plausible to assume that if users view OMC to have a more extraordinary reputation, they will perceive OMC as trustworthy and useful. Therefore, this study hypothesized that:

**H17.**  *Cognitive reputation will have a significant positive effect on trust*.

**H18.**  *Cognitive reputation will have a significant positive effect on perceived usefulness*.

### 2.4. The OMC Acceptance Model

**Figure 1:**
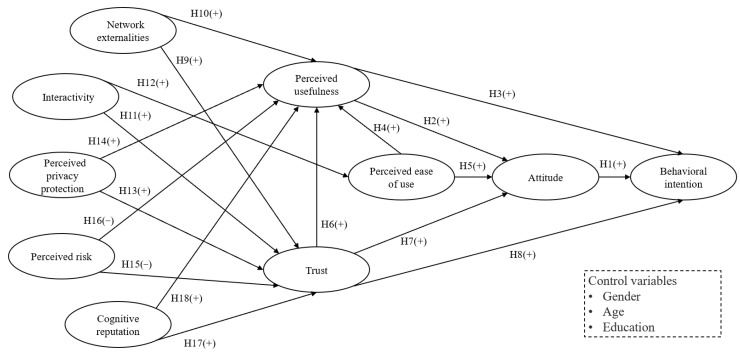
The proposed OMC acceptance model and the research hypotheses.

Based on the specification of the constructs, the OMC acceptance model was developed (see Figure 1).

## 3. Methodology

The purpose of this study was to examine the relationship between trust and its antecedent variables on healthcare consumers’ intention to use OMC. To achieve this, a quantitative and online survey-based study was conducted. This research method was appropriate for exploring the predictive relationships between variables and allowed for confirmatory findings [73]. An online survey was used to collect data due to its convenience, anonymity, and ability to reach a large population [74]. This method was suitable for answering research questions on self-reported beliefs and behaviors [75]. Overall, the combination of a quantitative research method and online survey allowed for the efficient collection and analysis of large amounts of data on healthcare consumers’ attitudes and orientations.

### 3.1. Measurement Instrument

This study utilized a well-designed three-part questionnaire to collect data from participants. The first section provided an overview of the study’s setting and objectives, while the second section collected demographic information, such as age and gender, and OMC experience. The final section extracted participants’ opinions on OMC adoption using ten constructs outlined in the study model. Previously validated scales from the literature were used to measure these constructs, and all variables had three or more measurement items on a seven-point Likert scale (see Appendix A). Response biases were controlled through procedural and statistical measures, such as independent examination of the questionnaire by researchers and preliminary study, with reference to [76]. The survey took approximately five minutes to complete, and the preliminary study results suggested the questionnaire’s validity.

### 3.2. Data Collection Procedure

This research got the Institutional Review Board approval from the first author’s institution (approval number: 201916). An online survey (via the Star Customer Questionnaire platform) with a self-administered questionnaire containing the three sections mentioned above was created to collect empirical data during the COVID-19 pandemic. All responses were completely anonymous. The data collected were stored on a password-protected computer accessible only by the research team. Informed consent was obtained from all participants prior to data collection.

The sample of this study was targeted at healthcare consumers, including ordinary people with the potential need for medical consultation and patients. Inclusion criteria include: (a) China residency, (b) at least 18 years of old, (c) no cognitive impairment, (d) being able to comprehend Chinese, and (e) being willing to participate in the study. Participants were recruited using convenient and snowball sampling techniques by posting invitation messages on WeChat groups, requesting group members to participate, and spreading the invitation to others. The snowball sampling technique was utilized because it might help locate hidden populations [77], mitigating non-response bias.

### 3.3. The Study Sample

The study sample size was determined using an a-priori sample size calculator for structural equation models [78]. Assuming an effect size of 0.3, a statistical power level of 0.8, 10 latent variables with 40 observed variables (i.e., indicators), and a probability level of 0.05, it can be calculated that a sample of 190 subjects is required for modeling structure and detecting effect, which also fulfills the ten-times rule. A total of 381 participants were enrolled in this study. After removing the invalid entries, 260 valid responses were obtained, which was considered sufficient for the following data analysis. The study participants came from diverse domains, education levels, and ages. Table 2 displays the demographic information of the participants. Among them, gender, age, and education were set as control variables in the model examination.

## 4. Data Analysis and Results

IBM SPSS Statistics version 26 (IBM Corp., Armonk, NY, USA) was employed to reveal the nature of the data by producing descriptive statistics, including mean, standard deviation (SD), 95% confidence interval (CI), skewness, and kurtosis, on the measurements. On the seven-point Likert-type scale, participants rated their behavior intention at an average of 4.82 (SD = 0.79), indicating a trend of spontaneous usage behavior. Other constructs averaged between 4.27 and 4.96. The construct’s descriptive statistics are displayed in Appendix B.

In addition, SmartPLS version 3 was used for measuring and assessing the structural model. This study utilized the partial least squares structural equation modeling (PLS-SEM) approach to evaluate the proposed model. PLS-SEM was considered an appropriate analysis method for this study because it enables simultaneous estimation of relationships between multiple independent and latent dependent variables [79]. It is also a suitable method for both reflective and formative constructs and can be used for multivariate normality and small sample sizes [80]. Moreover, PLS-SEM is well-suited for exploratory research and emphasizes prediction [79], which is one of this study’s primary aims.

### 4.1. Measurement Model Assessment

#### 4.1.1. Reliability, Convergent Validity, and Discriminant Validity

Anderson and Gerbing [81] suggested that items be examined first for reliability and then for various degrees of statistical validity, such as convergent and discriminant validity. Table 3 contains Cronbach’s alpha, composite reliability, average variance extraction (AVE), and the Fornell-Larcker test of the constructs. Cronbach’s alpha values of the constructs were between 0.80 and 0.93, suggesting that the internal consistency of the measurement items had been attained. The composite reliability values were between 0.87 and 0.95, indicating that the model’s internal consistency and reliability are acceptable [82]. All the AVE values exceeded 0.5, indicating that the model exhibited good convergent validity [82,83]. The Fornell-Larcker criterion was also satisfied, as indicated by the square root of the AVE values of each construct being larger than the correlation coefficients between other constructs, demonstrating good discriminant validity [84]. In addition, the heterotrait–monotrait correlation ratio (HTMT) ratios also revealed satisfactory discriminant validity (see Table 4) [85]. All the outer loadings of the items were more than 0.7 (except that NE4 and PEOU4, which are slightly less than 0.7) and greater than their cross-loadings (see Appendix C), showing that the measurement items were reliable and valid [82,83]. Consequently, the assessment of the measurement model concluded that the measurement’s overall reliability and validity were adequate.

#### 4.1.2. Multicollinearity and Common Method Bias

Multicollinearity can affect parameter estimation accuracy in structural models when multiple exogenous variables predict endogenous variables. To detect multicollinearity, the variance inflation factor (VIF) was calculated in this study. According to Hair et al. [82], a VIF value between 0.2 and 5 indicates that the model is not multicollinear. This study’s inner (factor-level) VIF values ranged from 1.00 to 2.00, indicating the absence of multicollinearity in the structural model.

### 4.2. Structural Model Assessment

Standardized Root Mean Square Residuals (SRMR) and Normative Fit Index (NFI) were used to measure the PLS-SEM model fit. This study’s SRMR value was 0.09, whereas the NFI value was 0.68, suggesting that the model fit is within the acceptable range. Moreover, the cross-validation redundancy index (Q2) was derived for the structural model. The findings revealed that all Q2 values were larger than 0, suggesting the model exhibited predictive validity.

**Figure 2:**
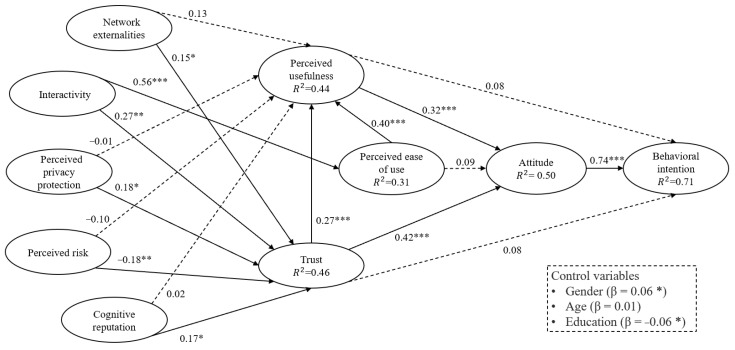
Model testing results from the PLS-SEM analysis, * *p* < 0.05, ** *p* < 0.01, *** *p* < 0.001.

This study estimated the path coefficients for each hypothesized path and assessed their statistical significance using 5000 bootstrap subsamples of random observations from the given dataset. Detailed results can be found in Appendix D. To determine the total variance that could be explained using the predictor constructs, the endogenous constructs’ coefficients of determination (i.e., the adjusted R^2^ values) were calculated. As shown in Figure 2, the R^2^ values of BI, ATT, TRU, PU, and PEOU are 0.71, 0.50, 0.46, 0.44, and 0.31, respectively, indicating that the proposed research model possesses remarkable explanatory and prediction power. The path coefficient estimation results of the OMC acceptance model are summarized in Table 5. Our study demonstrated that 11 of the 18 hypotheses were supported. Specifically, BI was positively and significantly affected by ATT (β = 0.74, *p* < 0.001), supporting H1. ATT was positively and significantly affected by PU (β = 0.32, *p* < 0.001) and TRU (β = 0.42, *p* < 0.001). Therefore, H2 and H7 hold. PU was positively and significantly affected by TRU (β = 0.27, *p* < 0.001) and PEOU (β = 0.40, *p* < 0.001). Therefore, H4 and H6 hold. PEOU was positively and significantly affected by INT (β = 0.56, *p* < 0.001), so H12 holds. TRU was positively affected by NE (β = 0.15, *p* < 0.05), INT (β = 0.27, *p* < 0.01), PPP (β = 0.18, *p* < 0.05), and CR (β = 0.17, *p* < 0.05), and negatively affected by PR (β = −0.18, *p* < 0.01). Thus, H9, H11, H13, H15 and H17 hold. The structural model assessment results are depicted in Figure 2.

## 5. Discussion

This study was performed to build a theoretical model (i.e., the OMC acceptance model) that describes how healthcare consumers’ intentions to use OMC are developed. Based on the technology acceptance model as a theoretical base, this study provides a structural model to examine healthcare consumers’ adoption of OMC by including trust and its antecedent variables. A total of 10 constructs and 18 hypotheses were set in the model to explore the interacting connections between the constructs. As a result, 11 of the hypotheses were supported by the study results, and the OMC acceptance model was able to explain 50% of the variance in attitude and 71% of the variance in behavioral intention.

Similar to the conclusion of TAM [40,41], our study found that PU had a significant effect on ATT, whereas PEOU significantly affects PU. Moreover, our study found that PEOU significantly affected ATT and BI through PU (total effect for ATT = 0.22, *p* < 0.001; for BI total effect = 0.19, *p* < 0.001), emphasizing the importance of PEOU in developing users’ OMC acceptance. It is shown that healthcare consumers’ attitude towards OMC significantly predicted their behavioral intention to use it. However, contrary to expectations, this study found that the direct path from PEOU to ATT was not significant. While the direct effect of PU on BI was not significant, the total effect of PU on BI was significant and mediated by ATT (total effect = 0.32, *p* < 0.001). This indicates that healthcare consumers were more likely to develop positive attitudes and behavioral intentions toward OMC if they believed that using OMC would improve their current health.

With regard to trust, our results showed that TRU had a relatively larger impact on ATT (β = 0.42) compared to PU (β = 0.32). Although TRU did not show a significant direct effect on BI, its total effect was significant and considerable (total effect = 0.48, *p* < 0.001). This shows that trust plays a vital role in influencing healthcare consumers’ acceptance of OMC [86]. This could be because trust may help reduce the complexity and uncertainty associated with OMC [8,87], making healthcare consumers believe that OMC is useful and have a positive attitude, thereby increasing users’ intention to use OMC.

Regarding the antecedent variables of trust, this research found that NE, PPP, and CR had significant positive effects on TRU, consistent with previous studies [61,70,88]. Additionally, NE (total effect = 0.11, *p* < 0.01) and PPP (total effect = 0.08, *p* < 0.05) significantly affected BI through mediators. This implied that NE and PPP substantially impacted user acceptance. This may be due to healthcare consumers’ belief that OMC will become more trustworthy when the user network of OMC is larger, and their privacy can be protected, thus helping to increase usage intention. Moreover, INT had a significant positive effect on TRU and PEOU, consistent with previous findings [10,89,90]. This result indicates that users who perceive OMC to facilitate mutual communication are more likely to perceive the technology as simple and trustworthy. The total effect of INT on BI (total effect = 0.24, *p* < 0.001) was also significant, implying that INT may have played a relatively important role in adopting OMC. In other words, if healthcare consumers believe that OMC can facilitate mutual communication, they will be more likely to develop trust and perceive the technology as easy, thereby increasing their willingness to use OMC. Furthermore, consistent with previous research [21,91], PR had a significant negative impact on TRU. Due to health problems, health consumers will pay more attention to risks, trying to avoid them. Our study findings also demonstrated significant total effects of PR on PU, ATT, and BI. It shows that if the risk of OMC is not controlled, even if OMC has many advantages, healthy consumers will reduce their evaluation and willingness to use OMC [3]. Therefore, it is necessary to reduce the perceived risk to increase trust and incentivize users to adopt OMC.

## 6. Conclusions

The study investigated the factors that influence healthcare consumers’ acceptance of OMC, a new technology that faces challenges in acceptance. The proposed model integrated the TAM with trust and its antecedent variables to explain OMC acceptance. The results showed that ATT influenced users’ BI. PU, PEOU, and TRU significantly affected BI through the mediation of ATT. PR, PPP, NE, CR, and INT all have a direct impact on TRU. The proposed model explained 50% and 71% of the variance in healthcare consumers’ attitude and behavior intention, respectively.

This study has theoretical and practical implications. The theoretical implications of this study relate to the novel integration of trust and its antecedent variables in examining OMC acceptance among healthcare consumers. While prior research has focused on key factors such as perceived ease of use and perceived usefulness, trust has been overlooked in the OMC context, despite its crucial role in medical consultations. Nevertheless, trust is essential in medical consultations. It has been found to influence users’ behavior online [22], especially in online medical settings [30]. This study sheds light on the influence of perceived risk, perceived privacy protection, network externalities, cognitive reputation, and interactivity on trust. These findings offer unique insights into understanding users’ acceptance of OMC from the trust perspective.

On practical grounds, the findings have practical implications for policymakers, OMC developers, and OMC platform providers in China and other countries. Understanding the factors that influence healthcare consumers’ acceptance of OMC is crucial for developing and implementing OMC services. OMC platform providers can take measures to increase healthcare consumers’ confidence, such as strengthening management and improving service quality to reduce potential hazards. Improving the usefulness and ease of use of OMC platforms is also necessary, for example, by creating a prompt inquiry after one week to determine whether healthcare consumers’ needs have been met [92] and designing a more intimate mode for the elderly [93]. Policymakers can also introduce policies to encourage the use of OMC, such as incentivizing high-quality physicians to participate in OMC [94].

In conclusion, this study sheds light on the crucial role of trust and its antecedent variables in healthcare consumers’ acceptance of OMC, providing valuable insights for OMC researchers, providers, and policymakers. This study has the following limitations. First, similar to previous studies on user acceptance, this study relied on behavioral intention as a proxy for acceptance. Although research in other fields has demonstrated a strong relationship between users’ behavioral intention and usage behavior [95], the link between intention and actual usage behavior needs further investigation. Second, this study only evaluated ten constructs. Other factors, such as subjective norm [96] and perceived severity [97], are suggested to be examined with a larger sample size. Third, this study was conducted in China. Further research should be conducted in other countries or regions to cross-validate the results. In future research, we plan to use methods beyond questionnaires, such as semi-structured interviews, to collect data and recruit larger samples for validation purposes.

## Figures and Tables

**Table 1:** Literature review in the present study context.

Literature	Study Sample	Independent Variable	Dependent Variable	Study Setting
[26]	4231 physicians	Online reputation, offline reputation, online effort	E-consultation choice	Commercial online consultation platforms
[27]	413 patients	Push factors (inconvenience, perceived risk), pull factors (ubiquitous care, opportunity of alternatives), trust, switching costs, habit, inertia	Switching intentions	e-health consultations platforms
[28]	1264 patients	Perceived health status, patient activation, Internet health information seeking, ease of Internet access	Communication with doctors on the Internet	Email, social media, and mobile app
[29]	907 physicians	Online service reviews, offline service reviews	Number of patients’ telephone consultations	Online health community
[8]	543 university students	Perceived risk, perceived benefit, trust in providers, subjective norm, offline habit	Adoption of OHCS	Online health consultation services platforms
[30]	339 orthopedic patients	Perceived value, perceived trust	Intention to consult	Online medical community
[14]	378 patients	Performance expectancy, effort expectancy, social influence, attitude toward using technology, behavioral intention	Usage behavior	Online health communities
[31]	486 healthcare consumers	Tangible attributes of health information providers, intangible attributes of health information providers, consumer needs for health information	Acceptance of online health communication	Social networking sites
[32]	2309 physicians	Service provision, service pricing	Patient satisfaction	Online health communities
[33]	35,597 voice-based medical services provided by physicians	Speech rate, average spectral centroid, professional capital	Patient satisfaction	Online health consultation
[34]	338 healthcare consumers	Perceived benefits, perceived costs, sunk costs, health service habits, transition costs, privacy protection beliefs	Use intention	Online health services platforms
[35]	8401 physicians	Online rating, activeness	The number of patients	Online health community
[36]	292 rural end-users	Age, gender, education, monthly family expenditure, attitude toward the system, perceived system effectiveness, cellphone ownership, advertisements, social reference	eHealth acceptance	Portable health clinic
[37]	5521 physicians	Negative sentiment, readability, depth, spelling, information helpfulness	Treatment choice	Physician rating websites
[38]	831 physicians	Social ties, knowledge ties	Patient selection (online selection and offline selection)	Online consultation platform
[39]	578 patients	Performance expectancy, effort expectancy, social influence, facilitating condition, perceived risk, trust, behavioral intention	Adoption	Digital health consultation apps

**Table 2:** Demographic statistics of the study sample (*n* = 260).

Item		*n*	%
Gender			
	Male	69	26.54
	Female	191	73.46
Mean age (SD)			39.07 y/o (10.18)
Education level			
	High school and below	24	9.23
	College or equivalent degree	30	11.54
	Undergraduate	158	60.77
	Postgraduate	48	18.46
OMC functions that participants concerned ^†^			
	Gain healthcare knowledge	82	31.54
	Efficient, professional, and reliable emergency medical service	12	4.62
	Make appointments on the platform	59	22.69
	Consult authoritative experts	47	18.08
	Diagnose the symptoms and further discuss the disease conditions	33	12.69
	Acquire more private medical consultation	8	3.08
	Buy prescription drugs	7	2.69
	Communicate with peers in the doctor-patient community on the platform	15	5.77
	Serve as a supplementary measure to the offline medical consultation	21	8.08
	Others (e.g., viewing medical records)	1	0.38

^†^ Multiple-choice question.

**Table 3:** Cronbach’s alpha, composite reliability, AVE, and the Fornell-Larcker test of the constructs.

	Cronbach’s Alpha	Composite Reliability	AVE	Fornell-Larcker Criterion									
				ATT	BI	CR	INT	NE	PEOU	PPP	PR	PU	TRU
ATT	0.89	0.93	0.82	0.90									
BI	0.86	0.92	0.78	0.84	0.88								
CR	0.85	0.90	0.68	0.65	0.56	0.83							
INT	0.90	0.93	0.77	0.63	0.65	0.63	0.88						
NE	0.88	0.90	0.61	0.69	0.68	0.55	0.53	0.78					
PEOU	0.80	0.87	0.63	0.45	0.41	0.39	0.56	0.39	0.79				
PPP	0.93	0.95	0.79	0.47	0.48	0.52	0.54	0.39	0.30	0.89			
PR	0.81	0.88	0.71	−0.25	−0.28	−0.18	−0.09	−0.16	−0.07	−0.14	0.84		
PU	0.87	0.91	0.66	0.60	0.55	0.41	0.52	0.44	0.57	0.32	−0.23	0.81	
TRU	0.80	0.88	0.72	0.63	0.59	0.56	0.58	0.49	0.42	0.51	−0.28	0.53	0.85

Note: ATT, attitude; BI, behavioral intention; CR, cognitive reputation; NE, network externalities; PEOU, perceived ease of use; PPP, perceived privacy protection; PR, perceived risk; PU, perceived usefulness; TRU, trust; INT, interactivity.

**Table 4:** HTMT criterion results.

	ATT	BI	CR	INT	NE	PEOU	PPP	PR	PU	TRU
ATT										
BI	0.96									
CR	0.74	0.65								
INT	0.70	0.73	0.70							
NE	0.76	0.75	0.61	0.55						
PEOU	0.53	0.49	0.48	0.65	0.43					
PPP	0.51	0.52	0.56	0.58	0.40	0.34				
PR	0.28	0.32	0.20	0.10	0.22	0.17	0.15			
PU	0.68	0.63	0.47	0.58	0.47	0.68	0.34	0.26		
TRU	0.74	0.70	0.64	0.67	0.54	0.52	0.58	0.32	0.62	

Note: ATT, attitude; BI, behavioral intention; CR, cognitive reputation; NE, network externalities; PEOU, perceived ease of use; PPP, perceived privacy protection; PR, perceived risk; PU, perceived usefulness; TRU, trust; INT, interactivity.

**Table 5:** Results summary.

Hypothesis	Path	Beta	*t* Statistic	Decision
H1	ATT → BI	0.74 ***	11.58	Yes
H2	PU → ATT	0.32 ***	4.03	Yes
H3	PU → BI	0.08	1.41	No
H4	PEOU → PU	0.40 ***	6.37	Yes
H5	PEOU → ATT	0.09	1.44	No
H6	TRU → PU	0.27 ***	3.63	Yes
H7	TRU → ATT	0.42 ***	6.54	Yes
H8	TRU → BI	0.08	1.19	No
H9	NE → TRU	0.15 *	2.07	Yes
H10	NE → PU	0.13	1.79	No
H11	INT → TRU	0.27 **	2.97	Yes
H12	INT → PEOU	0.56 ***	10.94	Yes
H13	PPP → TRU	0.18 *	2.37	Yes
H14	PPP → PU	−0.01	0.20	No
H15	PR → TRU	−0.18 **	3.04	Yes
H16	PR → PU	−0.10	1.59	No
H17	CR → TRU	0.17 *	2.25	Yes
H18	CR → PU	0.02	0.27	No

Notes: ATT, attitude; BI, behavioral intention; CR, cognitive reputation; NE, network externalities; PEOU, perceived ease of use; PPP, perceived privacy protection; PR, perceived risk; PU, perceived usefulness; TRU, trust; INT, interactivity. * *p* < 0.05; ** *p* < 0.01; *** *p* < 0.001.

## Data Availability

The datasets used in this study are available from the corresponding author upon reasonable request.

## Appendix A

**Table A1:** Constructs and measurement items in the questionnaire.

Construct	Item	Content
Attitude (ATT) [98,99]	ATT1	Using OMC is (would be) a good idea.
	ATT2	I like (would like) using OMC.
	ATT3	It is desirable for me to learn how to use OMC.
Behavioral intention (BI) [98,100]	BI1	I plan to use OMC frequently.
	BI2	I intend to use OMC when needed.
	BI3	If feasible, I will use OMC.
Cognitive reputation (CR) [101,102]	CR1	OMC service is provided by prestigious hospitals.
	CR2	OMC platforms’ partners have a good reputation.
	CR3	I think the reputation of the OMC platform is the main reason I am considering using it.
	CR4	I think the main reason for using an OMC platform is its good reputation.
Network externalities (NE) [103]	NE1	I think a good number of people use OMC platforms.
	NE2	I think most people are using OMC.
	NE3	I think there will still be many people joining OMC platforms.
	NE4	I think many friends around me use OMC.
	NE5	I think most of my friends are using OMC.
	NE6	I anticipate many friends will use OMC in the future.
Perceived ease of use (PEOU) [40]	PEOU1	It is easy for me to learn how to use OMC.
	PEOU2	I find it easy to get OMC to do what I want (e.g., inquire about personal health information and communicate with professionals).
	PEOU3	I think OMC are complicated to understand.
	PEOU4	Interacting with the OMC platforms requires only a little of my mental effort.
Perceived privacy protection (PPP) [104]	PPP1	If the OMC platform wants to use my personal information for some unauthorized purposes, it would ask for my permission in advance.
	PPP2	If the OMC platform wants to share my personal information with others, it would ask for my permission in advance.
	PPP3	I think the OMC platform would not sell users’ personal information to others without users’ permission.
	PPP4	I think the OMC platform has taken strict measures and established mechanisms to protect users’ personal information.
	PPP5	I think the OMC platform has provided good information protection for users.
Perceived risk (PR) [102]	PR1	Using OMC would involve more health risks when compared with offline medical consultation.
	PR2	Using OMC would involve more financial risk when compared with offline medical consultation.
	PR3	I think the overall risk of OMC is higher than offline medical consultation.
Perceived usefulness (PU) [40,98,102]	PU1	Using OMC enhances my effectiveness in medical consultation.
	PU2	Using OMC enhances the effect of medical consultation.
	PU3	Using OMC provides me access to useful health information.
	PU4	Using OMC improves my health condition.
	PU5	OMC enables me to accomplish a medical consultation more quickly than offline medical consultation.
Trust (TRU) [105]	TRU1	In general, OMC (platforms/ healthcare professionals) are trustworthy.
	TRU2	OMC (platforms/ healthcare professionals) give the impression that they keep promises and commitments.
	TRU3	I believe that OMC (platforms/ healthcare professionals) have my best interests in mind.
Interactivity (INT) [106]	INT1	OMC platforms are effective in gathering users’ feedback.
	INT2	OMC platforms facilitate two-way communication between users and healthcare professionals.
	INT3	OMC platforms make me feel they want to listen to their users.
	INT4	OMC platforms allow users to talk back and provide feedback.

Note: PEOU3 was the reversed question for experimental design. The item’s entry was corrected in data analysis to reflect the original definition of the corresponding construct. The original questionnaires were in Chinese. They were translated into English and reviewed to ensure the translation was correct.

## Appendix B

**Table A2:** Descriptive statistics of the constructs.

Construct	Mean	SD	95% CI	Skewness	Kurtosis
Attitude	4.85	0.87	[4.75, 4.96]	0.50	2.14
Behavioral intention	4.82	0.79	[4.73, 4.92]	0.70	2.81
Cognitive reputation	4.89	0.87	[4.78, 5.00]	0.67	0.34
Interactivity	4.89	0.80	[4.79, 4.99]	0.81	1.35
Network externalities	4.72	0.71	[4.63, 4.81]	0.85	1.71
Perceived ease of use	4.89	0.87	[4.79, 5.00]	0.42	0.79
Perceived privacy protection	4.49	1.08	[4.35, 4.62]	0.38	0.52
Perceived risk	4.27	0.95	[4.15, 4.38]	−0.04	1.27
Perceived usefulness	4.96	0.91	[4.84, 5.07]	0.10	1.44
Trust	4.43	0.79	[4.34, 4.53]	1.36	2.85

## Appendix C

**Table A3:** Outer loadings and cross-loadings for the measurement items.

	ATT	BI	CR	INT	NE	PEOU	PPP	PR	PU	TRU
ATT1	0.91	0.74	0.63	0.59	0.65	0.47	0.44	−0.21	0.57	0.62
ATT2	0.88	0.73	0.52	0.48	0.59	0.38	0.39	−0.22	0.55	0.56
ATT3	0.92	0.80	0.61	0.62	0.64	0.38	0.46	−0.24	0.51	0.54
BI1	0.71	0.82	0.41	0.45	0.52	0.31	0.42	−0.28	0.48	0.53
BI2	0.72	0.91	0.53	0.62	0.63	0.38	0.44	−0.20	0.48	0.48
BI3	0.78	0.92	0.55	0.64	0.64	0.39	0.41	−0.26	0.51	0.55
CR1	0.54	0.44	0.84	0.51	0.38	0.30	0.47	−0.22	0.30	0.48
CR2	0.62	0.55	0.89	0.61	0.49	0.32	0.58	−0.21	0.38	0.60
CR3	0.47	0.39	0.76	0.40	0.44	0.31	0.26	−0.04	0.30	0.30
CR4	0.49	0.47	0.81	0.52	0.53	0.38	0.35	−0.09	0.35	0.40
INT1	0.58	0.58	0.58	0.92	0.45	0.54	0.52	−0.08	0.50	0.52
INT2	0.62	0.61	0.55	0.91	0.52	0.53	0.51	−0.11	0.51	0.52
INT3	0.44	0.48	0.48	0.84	0.38	0.44	0.47	−0.07	0.37	0.49
INT4	0.55	0.59	0.59	0.84	0.52	0.44	0.40	−0.05	0.41	0.49
NE1	0.60	0.57	0.47	0.48	0.81	0.38	0.36	0.01	0.42	0.48
NE2	0.52	0.49	0.37	0.37	0.82	0.31	0.25	−0.15	0.33	0.37
NE3	0.61	0.66	0.57	0.56	0.84	0.38	0.35	−0.14	0.42	0.46
NE4	0.39	0.37	0.25	0.19	0.69	0.15	0.20	−0.19	0.19	0.18
NE5	0.47	0.43	0.32	0.26	0.72	0.18	0.29	−0.18	0.25	0.27
NE6	0.59	0.56	0.50	0.46	0.81	0.32	0.31	−0.19	0.33	0.40
PEOU1	0.32	0.27	0.25	0.42	0.32	0.83	0.21	0.08	0.45	0.25
PEOU2	0.44	0.40	0.37	0.54	0.37	0.91	0.32	−0.07	0.59	0.40
PEOU3	0.35	0.35	0.27	0.44	0.31	0.76	0.22	−0.15	0.34	0.30
PEOU4	0.30	0.25	0.35	0.34	0.21	0.65	0.18	−0.10	0.39	0.36
PPP1	0.33	0.29	0.44	0.37	0.27	0.29	0.80	−0.03	0.19	0.39
PPP2	0.40	0.37	0.43	0.43	0.31	0.25	0.88	−0.05	0.25	0.36
PPP3	0.42	0.41	0.45	0.47	0.32	0.23	0.90	−0.09	0.26	0.42
PPP4	0.45	0.50	0.49	0.54	0.39	0.28	0.92	−0.21	0.33	0.52
PPP5	0.48	0.51	0.50	0.56	0.40	0.29	0.93	−0.19	0.34	0.52
PR1	−0.19	−0.22	−0.10	−0.02	−0.08	−0.01	−0.08	0.82	−0.16	−0.18
PR2	−0.27	−0.29	−0.23	−0.14	−0.24	−0.15	−0.16	0.87	−0.24	−0.30
PR3	−0.15	−0.16	−0.09	−0.03	−0.04	0.02	−0.10	0.84	−0.15	−0.20
PU1	0.51	0.47	0.37	0.45	0.40	0.50	0.25	−0.19	0.83	0.43
PU2	0.40	0.33	0.26	0.35	0.26	0.44	0.17	−0.12	0.79	0.40
PU3	0.52	0.51	0.30	0.40	0.40	0.41	0.27	−0.18	0.86	0.48
PU4	0.55	0.52	0.36	0.47	0.39	0.46	0.31	−0.22	0.86	0.51
PU5	0.44	0.39	0.35	0.42	0.30	0.51	0.28	−0.19	0.72	0.33
TRU1	0.57	0.52	0.50	0.54	0.45	0.40	0.39	−0.24	0.54	0.86
TRU2	0.58	0.55	0.51	0.53	0.45	0.33	0.47	−0.32	0.42	0.88
TRU3	0.44	0.39	0.40	0.37	0.32	0.31	0.43	−0.14	0.38	0.79

Note: ATT, attitude; BI, behavioral intention; CR, cognitive reputation; NE, network externalities; PEOU, perceived ease of use; PPP, perceived privacy protection; PR, perceived risk; PU, perceived usefulness; TRU, trust; INT, interactivity.

## Appendix D

**Table A4:** Path coefficient estimation and bootstrapping results of the OMC acceptance model.

Path	Hypothesis (Supported?)	Path Coefficient	*t*-Value for Path Coefficient	Total Effect	*t*-Value for the Total Effect
ATT → BI	H1 (Yes)	0.74 ***	11.58	0.74 ***	11.58
CR → ATT		—	—	0.09	1.91
CR → BI		—	—	0.09	1.95
CR → PU	H18 (No)	0.02	0.27	0.07	0.92
CR → TRU	H17 (Yes)	0.17 *	2.25	0.17 *	2.25
NE → ATT		—	—	0.12 **	2.58
NE → BI		—	—	0.11 **	2.63
NE → PU	H10 (No)	0.13	1.79	0.17 *	2.37
NE → TRU	H9 (Yes)	0.15 *	2.07	0.15 *	2.07
PEOU → ATT	H5 (No)	0.09	1.44	0.22 ***	4.18
PEOU → BI		—	—	0.19 ***	4.47
PEOU → PU	H4 (Yes)	0.40 ***	6.37	0.40 ***	6.37
PPP → ATT		—	—	0.09 *	2.14
PPP → BI		—	—	0.08 *	2.00
PPP → PU	H14 (No)	−0.01	0.20	0.04	0.52
PPP → TRU	H13 (Yes)	0.18 *	2.37	0.18 *	2.37
PR → ATT		—	—	−0.12 **	2.97
PR → BI		—	—	−0.12 **	3.00
PR → PU	H16 (No)	−0.10	1.59	−0.15 *	2.20
PR → TRU	H15 (Yes)	−0.18 **	3.04	−0.18 **	3.04
PU → ATT	H2 (Yes)	0.32 ***	4.03	0.32 ***	4.03
PU → BI	H3 (No)	0.08	1.41	0.32 ***	3.76
TRU → ATT	H7 (Yes)	0.42 ***	6.54	0.51***	8.70
TRU → BI	H8 (No)	0.08	1.19	0.48 ***	6.93
TRU → PU	H6 (Yes)	0.27 ***	3.63	0.27 ***	3.63
INT → ATT		—	—	0.26 ***	4.69
INT → BI		—	—	0.24 ***	4.58
INT → PEOU	H12 (Yes)	0.56 ***	10.94	0.56 ***	10.94
INT → PU		—	—	0.30 ***	5.78
INT → TRU	H11 (Yes)	0.27 **	2.97	0.27 **	2.97

Notes: ATT, attitude; BI, behavioral intention; CR, cognitive reputation; NE, network externalities; PEOU, perceived ease of use; PPP, perceived privacy protection; PR, perceived risk; PU, perceived usefulness; TRU, trust; INT, interactivity. * *p* < 0.05; ** *p* < 0.01; *** *p* < 0.001.
